# Real-time polymerase chain reaction results of urogenital system samples and their relationship with other sexually transmitted diseases

**DOI:** 10.12669/pjms.42.2.13171

**Published:** 2026-02

**Authors:** Emel Caliskan

**Affiliations:** Emel Caliskan, MD Associate Professor Duzce University, Faculty of Medicine, Department of Medical Microbiology, Duzce, Turkiye

**Keywords:** Polymerase chain reaction, Urogenital system infection, Sexually transmitted infections

## Abstract

**Objectives:**

This study aimed to examine the results obtained by multiplex real-time polymerase chain reaction (PCR) for microorganisms in urogenital tract samples sent to the molecular microbiology laboratory than patients of sexually transmitted infections suspected. In addition, we evaluated them alongside HIV, HBV, HCV, and syphilis test results.

**Method:**

Urine, vaginal swab, and urethral discharge samples submitted to Duzce University, Medical Microbiology Laboratory between March 2023 to November 2023 were included in the cross-sectional study. Multiplex PCR was used to assess the positivity of *Ureaplasma urealyticum, Ureaplasma parvum, Mycoplasma hominis, Neisseria gonorrhea, Trichomonas vaginalis, Mycoplasma genitalium, Treponema pallidum, Haemophilus ducreyi*, and *Chlamydia trachomatis*. Additionally, the patients’ serological test results for HBV, HCV, HIV, and syphilis were examined retrospectively.

**Results:**

A total of one hundred patient samples were sent to the Molecular Microbiology Laboratory during the study period. Twenty-eight patients were female and 72 were male, and the mean age was 34.11±10.63 (19-72). A total of 109 positive pathogens were detected in 65 (65%) patients. The positivity rate in women was 75% and in men was 62%, and was statistically similar (p=0.191). The most common pathogen detected in women was *U. parvum*, and in men was *M. genitalium*. Thirteen patients were positive for HIV, three for syphilis, and two for HBV.

**Conclusion:**

Molecular microbiological methods may be useful in patients suspected of having a urogenital system infection when no pathogens are cultured or when the presence of multiple pathogens must be identified to guide treatment.

## INTRODUCTION

Urogenital tract infections are caused by many microorganisms, including mycoplasmas, ureaplasmas, chlamydiae, *N. gonorrhea, H. ducrei, T. pallidum*, and *T. vaginalis*. These sexually transmitted infections (STIs), in addition to being urogenital diseases, also play a significant role in male and female infertility.^1^,^2^

More than one million curable STIs are acquired every day worldwide in people 15-49 years old, the majority of which are asymptomatic. More than 30 types of bacteria, viruses, and parasites are known to be transmitted through sexual contact. The eight most common STIs have been associated with these pathogens. Four of these are treatable: syphilis, gonorrhea, chlamydia, and trichomoniasis, while the other four are hepatitis B virus, herpes simplex virus (HSV), HIV, and human papillomavirus (HPV).^3^

Ureaplasma species and *M. hominis* can be found as commensals in the lower genital tract of many healthy adults, and their role in causing urethritis remains controversial.^4^ Therefore, routine laboratory tests are not performed, but studies using real-time polymerase chain reaction (PCR) have shown different rates of positivity have been detected in patients with urethritis.^4^-^6^

Sexually transmitted infections can occur alone or in combination with multiple agents. When a sexually transmitted agent is detected in patients, pre-exposure prophylaxis (PrEP) to prevent HIV transmission is becoming increasingly widespread globally.^7^ Therefore, it is important for laboratories to identify all these agents.

This study aimed to examine the multiplex PCR results obtained from patient samples sent to the molecular microbiology laboratory with urogenital tract infection preliminary diagnosis and to evaluate these results together with serological test results for Human Immunodeficiency Virus (HIV), Hepatitis B Virus (HBV), Hepatitis C Virus (HCV), and syphilis.

## METHODOLOGY

Laboratory results of all patients whose urine, vaginal swab, and urethral discharge samples were sent to Duzce University, Medical Microbiology Laboratory from clinics with a preliminary diagnosis of urogenital tract infection between March 2023 to November 2023 were retrospectively analyzed in this cross-sectional study. The samples were evaluated for positivity for *U. urealyticum, U. parvum, M. hominis, N. gonorrhea, T. vaginalis, M. genitalium, T. pallidum, H. ducreyi*, and *C. trachomatis* using the Multiplex PCR method (Bosphore STDs Real-Time PCR Panel Kit, Anatolia, Turkey).

The patients’ anti-HIV, HBsAg, and anti-HCV results, obtained using Chemiluminescence Microparticle Immunoassay (CMIA) using Architect kits on the Architect i2000sr instrument (Abbott, Germany), and confirmation results using the PCR method (Anatolia, Turkey), were analyzed. The results of syphilis serological tests assessed with the Treponema Pallidum Hemagglutination Assay (TPHA, Biomedica diagnostic, Malta) were also examined.

### Ethical Approval:

Ethical approval for our study was obtained from the Non-Interventional Clinical Research Ethics Committee of Duzce University (Decision No. 2025/198; Dated: July 28, 2025).

### Statistical Analysis:

Statistical Package for the Social Sciences software for Windows, version 22.0, was used for the statistical analysis (SPSS, Chicago, IL, USA). Appropriate descriptive statistics were calculated according to the type of data. The relationships between categorical variables were analysed by Chi-square, Fisher’s Exact tests. p<0.05 was considered statistically significant.

## RESULTS

**Fig.1:**
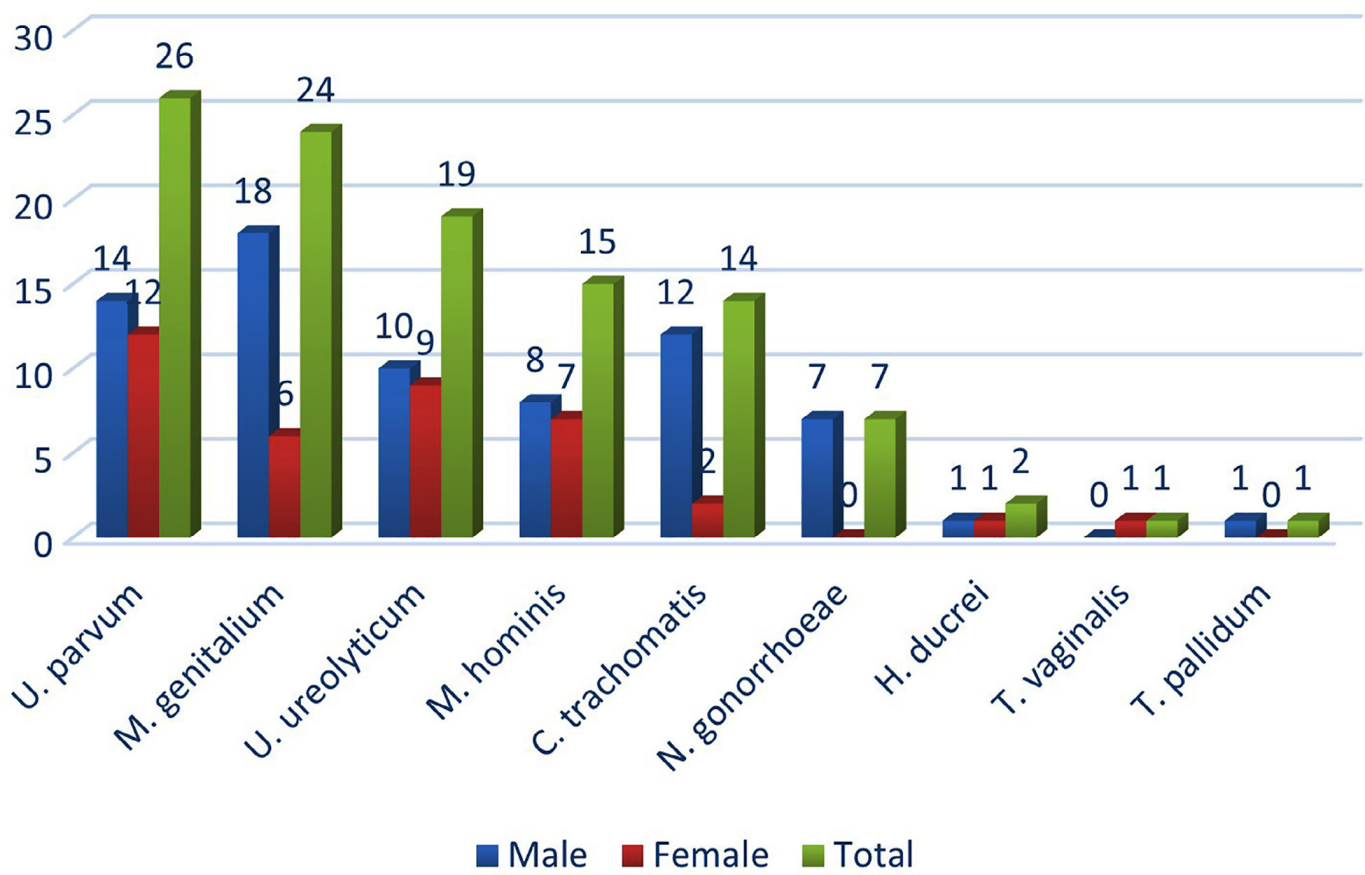
Positive detected microorganisms and their distribution by gender (n) .

During the study, a total of one hundred patient samples were sent to the Molecular Microbiology Laboratory for analysis with the STDs Real-Time PCR Panel Kit. Twenty-eight patients were female and 72 were male, with a mean age of 34.11±10.63 (range, 19-72). A total of 109 positive pathogens were detected in 65 (65%) patient samples, of which a single pathogen was isolated in 35 (54%) and multiple pathogens in 30 (46%). The positivity rate was 75% in women (n=21) and 62% in men (n=45), with no statistically significant difference (p=0.191). The most common pathogen detected in women was *U. parvum*, and in men, *M. genitalium*. *N. gonorrhoeae* positivity was detected only in male patients (10%) (Fig.1).

Of the patients included in the study, 13 were HIV positive, three were syphilis positive, and two were HBV positive. No statistically significant difference was found between those with positive and negative urogenital system PCR results in terms of syphilis, HBV, and HIV co-infection (p=0.140, p>0.999, P=0.665, respectively) (Table-I).

**Table-I:** Patients’ STDs Real-Time PCR Panel results and HBV, HCV, HIV, syphilis serology results.

	STDs Real-Time PCR Panel				
	Positive (n=65)		Negative (n=35)		
	n	%	n	%	p value
Syphilis	1/41	2.43	2/13	15.38	0.140
HBV	1/50	2.00	1/19	5.26	>0.999
HIV	9/57	15.78	4/20	20	0.665
HCV	-	-	-	-	

## DISCUSSION

Sexually transmitted diseases require diagnosis and treatment due to their potential complications, such as public health, maternal and fetal health, and potential infertility.^1^,^8^ *M. genitalium* has emerged in recent years as a sexually transmitted infection agent. It is recognized as causing acute and chronic urethritis in male and is associated with complicated infections. In femlae, it is associated with cervicitis, infertility, endometritis and PID (Pelvic Inflammatory Disease).^9^ *U. parvum* is a agent reported to be associated with infertility in female and male.^10^,^11^ *C. trachomatis* is the most frequently reported sexually transmitted bacterial infection worldwide.^12^ *C. trachomatis*, when combined with viral agents such as various HPV genotypes, HIV, and HSV-2, can cause cervical cancer.^13^ *N. gonorrhoea*, the causative agent of gonorrhea, is the second most commonly reported cause of STIs worldwide and causes genital tract infections such as urethritis and epididymitis in men.^12^ HIV, HCV, and syphilis, which can cause sexually transmitted systemic infections, can be detected alongside other agents that cause urogenital tract infections, such as *N. gonorrhoea*, chlamydia, mycoplasmas, and ureaplasmas. The presence of untreated sexually transmitted infections can accelerate the progression of HIV, while HIV infection can complicate the diagnosis and treatment of other sexually transmitted diseases (Zhang et al.^14^, Ford et al.^15^ and Boujema et al^16^).

In our study, the most frequently detected agent in male using the STDs Real-Time PCR Panel Kit was *M. genitalium* (25%). In Karpuz et al.^4^’s study, which included symptomatic men, the *M. genitalium* positivity rate was 6.4%. Sarier et al.^5^ found this rate to be 12.3%, Moridi et al.^17^ 16.6%, and Maede et al.^18^ 17%. Since *M. genitalium* are resistant to common antibiotics and the high prevalence of *M. genitalium* in our region physicians should be careful in the treatment of genitourinary tract infections to the possible presence of *M. genitalium* agent and the antibiotics.

In our study, the most frequently detected agent in female was *U. parvum* (43%). Similar to our study, a study conducted in female in Italy found the most frequent *U. parvum* positivity rate at 38.3%, while in Japan, this rate was 13.8%.^19^,^20^ It has been reported that the pathogenic effect of *U. parvum* may be mediated by locally produced proinflammatory cytokines and, unlike other genital mycoplasmas, may be independent of sialidase.^21^ The different results between the studies may be due to various factors, such as the patient group selected, the method used, and the patients’ risky sexual behaviors. Especially in patients presenting with infertility, *U. parvum* should be considered in our region due to its high prevalence.

In our study, *C. trachomatis* positivity was found at a rate of 14%, while Kirkoyun Uysal et al.^17^ reported this rate as 11.1%, Lopez et al.^22^ reported 8%, Wang et al.^23^ reported 16.94%, and Sarier et al.^5^ reported 9.9%. The existence of screening programs in countries or the ability to diagnose with appropriate tests may account for these differences.

In our study, *N. gonorrhoea* positivity was detected in 7 (7%) patients, all of whom were male (10%). Kirkoyun Uysal et al.^12^ reported a positivity rate of 3.9% for *N. gonorrhoea*, 5.92% for Wang et al.^23^ 3.5% for Lopez et al.^19^ and 4.09% for Cai et al.^24^ These different rates may be due to varying screening programs across countries and differences in the patient populations included in the study.

In our study, syphilis, HIV, and HBV positivity were observed in both patients with and without STIs positivity detected with the STIs Real-Time PCR Panel, while HCV positivity was not observed. HIV positivity (15.78%) was significantly higher in the study group than syphilis (2.43%) and HBV (2%) infections. This may be due to the fact that samples from patients with symptoms of, or a history of suspected STIs were evaluated. In addition, the fact that other STIs are investigated in HIV-positive patients with the STIs Real-Time PCR Panel as a routine screening program in our hospital explains the high HIV positivity rate.

This study, which identified the causative agents of urogenital system infections using the PCR method, has guided clinicians by providing up-to-date regional data. Planning multicentre studies in this area will enable the determination of the species distribution of causative microorganisms and the establishment of empirical treatment.

### Limitations:

Since our study was conducted retrospectively, HIV, HCV, HBV, and syphilis tests could not be performed on all one hundred patients included in the study, and therefore limited data in the hospital information system could be accessed.

## CONCLUSIONS

*U. parvum* in female and *M. genitalium* in male should be considered in urogenital tract infections where the causative agent is not detected by standard culture methods. Investigating STIs using rapid and sensitive molecular methods will enable the determination of STIs prevalence in the population and the initiation of appropriate treatments. Considering the high rates of co-infections of HIV positivity and other STIs, screening for other STIs in HIV-positive patients may facilitate the treatment of these infections.
